# New method of sex estimation through osteometric analysis of the lower limb for the Portuguese population

**DOI:** 10.1007/s00414-026-03902-1

**Published:** 2026-07-08

**Authors:** Sofia N. Wasterlain, Ricardo R. Dias, Maria Teresa Ferreira

**Affiliations:** 1 Research Centre for Anthropology and Health, Department of Life Sciences, University of Coimbra, Calçada Martim de Freitas, Coimbra, 3000-456 Portugal; 2 Coimbra, Portugal; 3 Centre for Functional Ecology, Laboratory of Forensic Anthropology, Department of Life Sciences, University of Coimbra, Calçada Martim de Freitas, Coimbra, 3000-456 Portugal; 4 Departamento de Ciências da Vida, Calçada Martim de Freitas, Coimbra, 3000-456 Portugal

**Keywords:** Forensic anthropology, Biological profile, Metric analysis, 21st identified skeletal collection

## Abstract

To reconstruct the identity of an individual, sex assessment is an essential step in the estimation of the biological profile of a skeleton. For that purpose, forensic anthropologists apply both morphological and metric methods based in skeletal elements. In 2000, Wasterlain used a sample from the Identified Skeletal Collection of the University of Coimbra (19th -20th centuries) to develop a method to estimate sex on the basis of the limb bones metrics. In the present study, a sample of 202 individuals (92 males and 110 females) from the 21st Century Identified Skeletal Collection (University of Coimbra) was used to revise the Wasterlain method to better suit the contemporary Portuguese population. In all, 22 measurements were performed in the femur, tibia, calcaneus, and talus. Although the Wasterlain method has shown satisfactory results, new cut-off points and discriminant functions were developed to promote accurate sex classifications in the contemporary Portuguese population. Regarding the cut-off points, the femoral vertical head diameter, the bilateral width of the tibia, the length of the calcaneus, and the maximum length of the talus presented the best results for sex estimation (89.8%, 88.0%, 85.6%, 85.9%, respectively). Discriminant functions presented correct classification percentages varying between 78.7% and 91.5% for femur, 83.7% and 93.0% for tibia, 84.9% and 86.6% for calcaneus, and 86.6% and 88.5% for talus. In sum, the new cut-off points and discriminant functions presented an overall good performance, constituting reliable methods to be applied in forensic cases involving individuals of Portuguese origin.

**Supplementary Information:**

The online version contains supplementary material available at https://doi.org/10.1007/s00414-026-03902-1.

## Introduction

Estimating sex from the analysis of human bones is crucial in the construction of the biological profile (sex, age at death, population affinity, and stature) of non-identified individuals. Researchers permanently seek to develop or improve methods that allow sex estimation with a high degree of certainty. Several studies have investigated which skeletal features present better capacity to differentiate between sexes [1–3]. It is unanimously agreed that the ossa coxae, given their role in pregnancy and childbirth, perform best in this task [3, 4]. Unfortunately, the irregular morphology and the compact-trabecular bone ratio of these skeletal elements turns them particularly vulnerable to the action of taphonomic agents [5]. Therefore, the creation of methods suitable for estimating sex from other bones is imperative. Traditionally, it was assumed by several researchers [e.g., 6, 7] that the skull was the second-best option for sex estimation. However, in recent years, the long bones, due to their high sexual dimorphism, have allowed the development of effective methods in various populations [5, 8–13]. Besides, long bones are generally used to estimate sex resorting to metric methods, which seem like a relatively straight-forward task with a faster learning curve, therefore giving more reliable results [11, 14, 15].

In Portugal, Wasterlain [16] used a sample from the Identified Skeletal Collection of the University of Coimbra [17] to develop a method to estimate sex on the basis of the limb bones (humerus, radius, femur, tibia, calcaneus, and talus) metrics. Although the cut-off points and discriminant functions developed by Wasterlain [16] are widely used in studies of past populations, their suitability for forensic cases needs verification, given the chronology of the sample used (19th -20th centuries). One of the most proven population changes over time is the increase in the individuals’ stature, mainly due to improvements in sanitary conditions, nutrition, and health of populations [18, 19]. Given that bone dimensions have changed over the past few decades [18, 19], and that these differences affect the accuracy of sex estimation [4, 20], then methods based on populations of distinct chronologies must be adapted to contemporary populations. In the present study, the Wasterlain [16] method was revised and adapted to be used in Portuguese forensic cases.

## Materials and methods

A sample of 202 adult individuals from the 21st Century Identified Skeletal Collection (CEI/XXI) [21], curated at the University of Coimbra, was used. Regarding the sex distribution, 92 were males (45.5%) and 110 were females (54.5%). In all, 22 measurements were performed in the femur, tibia, calcaneus, and talus. All measurements (Table 1) were taken in millimetres using a digital sliding calliper (Mitutoyo Absolute Digimatic), an osteometric board and a measuring tape, following Martin and Saller [22] and Holland [2]. Left-sided bones were preferentially measured. When the left bones were missing, fragmented, or affected by any pathology, right-sided bones were measured instead. In some cases, it was not possible to obtain all measurements mainly due to preservation/fragmentation issues. Given the exclusion criteria applied, the number of bones used in this study was the following: femur (*n* = 191); tibia (*n* = 198); calcaneus (*n* = 200); and talus (*n* = 199).

**Table 1:** Osteometric dimensions measured in the present study, following Martin and Saller (1957) and Holland (1991)

Bone	Measurement	Abbreviation	Source	Instrument
Femur	Maximum length	FML	Martin and Saller’s no. M-1	Osteometric board
	Physiological length	FPL	Martin and Saller’s no. M-2	Osteometric board
	Antero-posterior midshaft diameter	FAPMD	Martin and Saller’s no. M-6	Digital sliding calliper
	Transverse midshaft diameter	FTMD	Martin and Saller’s no. M-7	Digital sliding calliper
	Antero-posterior subtrochanteric diameter	FAPSD	Martin and Saller’s no. M-10	Digital sliding calliper
	Transverse subtrochanteric diameter	FTSD	Martin and Saller’s no. M-9	Digital sliding calliper
	Midshaft circumference	FMC	Martin and Saller’s no. M-8	Measuring tape
	Antero-posterior head diameter	FAPHD	Martin and Saller’s no. M-19	Digital sliding calliper
	Vertical head diameter	FVHD	Martin and Saller’s no. M-18	Digital sliding calliper
Tibia	Maximum length	TML	Martin and Saller’s no. M-1a	Osteometric board
	Natural length	TNL	Martin and Saller’s no. M-1b	Osteometric board
	Antero-posterior diameter at the nutrient foramen	TAPDNF	Martin and Saller’s no. M-8a	Digital sliding calliper
	Transverse diameter at the nutrient foramen	TTDNF	Martin and Saller’s no. M-9a	Digital sliding calliper
	Minimum circumference	TMC	Martin and Saller’s no. M-10b	Measuring tape
	Bilateral width	TBW	Holland no. M-1	Digital sliding calliper
Calcaneus	Maximum length	CML	Martin and Saller’s no. M-1	Digital sliding calliper
	Length	CL	Martin and Saller’s no. M-1a	
	Body length	CBL	Martin and Saller’s no. M-5	
	Medial Breath	CMB	Martin and Saller’s no. M-2	
Talus	Maximum length	TaML	Martin and Saller’s no. M-1	Digital sliding calliper
	Width	TaW	Martin and Saller’s no. M-2	
	Height	TaH	Martin and Saller’s no. M-3	

All measurements were performed by the second author (RD). A random subsample of 20 individuals was measured two weeks later to assess intra-observer error. The same subsample was also used to evaluate inter-observer error by an independent researcher (Beatriz Ribeiro) with similar anthropometric training.

Both intra- and inter-observer errors were calculated by using the relative technical error of measurement (rTEM), based on the technical error of measurement (TEM) [23]. TEM is an estimate of absolute precision, similar to the standard deviation of the magnitude of the error in the original measurement units (i.e., in mm), that allows anthropometrists to verify the accuracy degree when performing and repeating anthropometrical measurements (intra-observer) and when comparing their measurement with measurements from other anthropometrists (inter-observer). In practice, TEM is defined by the following equation:

$$\mathrm{TEM}=\sqrt{\frac{\sum\:{D}^{2}}{2N}}$$

where Σ𝐷2 represents the sum of the differences between the first and second measurements squared and N corresponds to the total number of individuals measured.

Using the TEM value, the relative TEM (rTEM) is then calculated using the following equation:

$$\mathrm{rTEM}=\frac{TEM}{\stackrel{-}{x}}$$

where $\:\stackrel{-}{x}\:$ refers to the average value of each variable. rTEM values up to 5% were considered acceptable.

In recent years, this index has been the most common way to express the error margin in anthropometry worldwide [e.g., 10, 12, 23]. For more details on this procedure, please refer to Perini et al. [23].

Bone measurements were used to estimate sex by applying the cut-off points and discriminant functions (DFs) developed by Wasterlain [16] (for these last ones, please refer to Supplementary Table 1). The agreement between the real sex and the estimated sex was assessed using Cohen’s Kappa test.

With the aim of improving the performance of Wasterlain’s method, making it more suited to the contemporary Portuguese population and forensic cases, new cut-off points and discriminant functions were developed. Cut-off points were obtained by calculating the midpoint between the average female and male values of each measurement, following Black [1]. The estimated sex is male or female if the value obtained through a given measurement is, respectively, greater or less than its cut-off point. Discriminant functions for each of the four bones were developed, whenever possible, by multivariate discriminant analysis [24], according to the following: *enter* procedure – combining all bone dimensions; *stepwise* procedure; *enter* procedure – combining the two bone dimensions whose new cut-off points present the best results; and, *enter* procedure – combining the two bone dimensions with the greatest representativeness.

The technical error of measurement was calculated using Microsoft Excel^®^. The remaining statistical analysis was conducted by using IBM SPSS Statistics for Windows^®^, Version 30.0. Armonk, NY: IBM Corp.

## Results

Relative TEM for intra- and inter-observer errors for each measurement are presented in Table 2. For intra-observer error, rTEM values vary between 0.1% (FML, FPL, and TML) and 2.9% (FAPSD). On the other hand, inter-observer error varies between 0.5% (FAPHD) and 5.9% (FAPSD).

**Table 2:** Relative TEM for intra- and inter-observer errors for each measurement

Measurement abbreviation	Relative TEM (%)	
	Intra-observer error	Inter-observer error
FML	0.1	0.6
FPL	0.1	0.7
FAPMD	1.2	2.2
FTMD	0.5	2.4
FAPSD	2.9	5.9
FTSD	2.6	5.8
FMC	1.5	1.2
FAPHD	0.4	0.5
FVHD	0.9	1.0
TML	0.1	0.7
TNL	0.5	0.8
TAPDNF	0.8	4.6
TTDNF	1.0	3.5
TMC	1.2	2.5
TBW	1.1	1.4
CML	0.8	1.6
CL	0.6	2.2
CBL	1.0	2.6
CMB	0.9	2.5
TaML	0.2	1.9
TaW	2.0	2.9
TaH	0.4	0.7

Table 3 shows the cut-off points proposed by Wasterlain [16] and the percentages of correct classification of sex obtained through their application to the present sample. For each bone, the measurements that gave overall best results in estimating the sex were FVHD (89.8%), TBW (86.2%), CMB (83.0%), and TaML (87.6%). In opposition, the worst results were obtained for FTMD (70.0%), TTDNF (77.7%), CBL (76.5%), and TaW (60.0%).

**Table 3:** Percentages of correct classification of sex through the application of cut-off points proposed by Wasterlain (2000)

Measurement abbreviation	Cut-off point (mm)	Males		Females		Total	
		*n*	%CC	*n*	%CC	*n*	%CC
FML	425.57	77	79.2	93	77.4	170	78.3
FPL	422.87	77	80.5	92	80.4	169	80.5
FAPMD	27.50	76	89.5	90	61.1	166	75.3
FTMD	26.07	77	81.8	93	58.1	170	70.0
FAPSD	27.29	82	61.0	103	88.3	185	74.7
FTSD	29.72	82	89.0	103	51.5	185	70.3
FMC	82.82	77	84.4	93	69.9	170	77.2
FAPHD	42.84	65	87.7	71	83.1	136	85.4
FVHD	43.23	69	92.8	83	86.7	152	**89.8**
TML	350.19	84	89.3	98	74.5	182	81.9
TNL	337.06	82	95.1	97	67.0	179	81.1
TAPDNF	32.47	87	90.8	109	70.6	196	80.7
TTDNF	23.56	87	73.6	109	81.7	196	77.7
TMC	69.29	87	73.6	109	84.4	196	79.0
TBW	68.18	66	93.9	79	78.5	145	**86.2**
CML	74.80	81	95.1	56	60.7	137	77.9
CL	70.44	81	64.2	57	96.5	138	80.4
CBL	53.34	82	95.1	57	57.9	139	76.5
CMB	39.58	85	90.6	85	75.3	170	**83.0**
TaML	50.30	88	89.8	102	85.3	190	**87.6**
TaW	39.02	73	100.0	75	20.0	148	60.0
TaH	28.99	91	89.0	102	80.4	193	84.7

Table 4 shows the percentages of correct classification of sex through the application of discriminant functions proposed by Wasterlain [16]. For the Wasterlain’s DFs, please refer to Supplementary Table 1. For each bone, the DF with the best performance were Femur DF7_W (90.9%), Tibia DF13_W (88.2%), Calcaneus DF21_W (85.2%), and Talus DF17_W (87.7%). The worst results in sex estimation were obtained with Femur DF8_W (86.8%), Tibia DF15_W (81.7%), Calcaneus DF20_W (75.2%), and Talus DF18_W (78.6%).

**Table 4:** Percentages of correct classification of sex through the application of discriminant functions proposed by Wasterlain (2000)

Bone and FD abbreviation from Wasterlain (2000)	Males		Females		Total	
	*N*	%CC	*N*	%CC	*N*	%CC
Femur DF7_W	61	98.4	66	83.3	127	**90.9**
Femur DF8_W	64	95.3	69	78.3	133	86.8
Femur DF9_W	64	93.8	71	84.5	135	89.2
Femur DF10_W	68	92.6	83	88.0	151	90.3
Femur DF11_W	68	91.2	83	88.0	151	89.6
Tibia DF13_W	65	96.9	78	79.5	143	**88.2**
Tibia DF14_W	65	95.4	78	79.5	143	87.5
Tibia DF15_W	87	87.4	108	75.9	195	81.7
Calcaneus DF19_W	78	67.9	53	92.5	131	80.2
Calcaneus DF20_W	78	57.7	54	92.6	132	75.2
Calcaneus DF21_W	78	83.3	54	87.0	132	**85.2**
Talus DF16_W	72	94.4	75	78.7	147	86.6
Talus DF17_W	89	92.1	85	83.3	191	**87.7**
Talus DF18_W	73	91.8	75	65.3	148	78.6

Although the Wasterlain [16] method has shown satisfactory results, new cut-off points and discriminant functions were developed in order to promote accurate sex classifications in the contemporary population. New cut-off points and the corresponding percentages of correct classification of sex are presented in Table 5. For each bone, the measurements that gave overall best results in estimating the sex were FVHD (89.8%), TBW (88.0%), CL (85.6%), and TaML (85.9%). On the other hand, the lowest percentages of correct classification were found for FTSD (73.7%), TTDNF (77.1%), CBL (82.1%), and TaW (81.1%).

**Table 5:** New cut-off points and respective percentages of correct classification of sex

Measurement abbreviation	Cut-off point (mm)	Males		Females		Total	
		*n*	%CC	*n*	%CC	*n*	%CC
FML	426.07	77	77.9	93	82.8	170	80.4
FPL	423.07	77	79.2	92	81.5	169	80.4
FAPMD	28.32	77	80.5	93	75.3	170	77.9
FTMD	26.79	77	64.9	93	74.2	170	69.6
FAPSD	26.53	82	72.0	103	82.5	185	77.3
FTSD	30.97	82	70.7	103	76.7	185	73.7
FMC	82.99	77	81.8	93	75.3	170	78.6
FAPHD	42.91	65	87.7	71	85.9	136	86.8
FVHD	43.25	69	92.8	83	86.7	152	**89.8**
TML	354.10	84	81.0	98	83.7	182	82.4
TNL	345.72	82	80.5	97	83.5	179	82.0
TAPDNF	33.40	85	87.1	108	83.3	193	85.2
TTDNF	23.61	87	72.4	109	81.7	196	77.1
TMC	68.66	87	83.9	109	81.7	196	82.8
TBW	68.81	66	92.4	79	83.5	145	**88.0**
CML	77.88	81	82.7	56	83.9	137	83.3
CL	67.36	81	85.2	57	86.0	138	**85.6**
CBL	56.06	82	81.7	57	82.5	139	82.1
CMB	40.43	85	81.2	85	84.2	170	82.7
TaML	50.87	89	85.4	103	86.4	192	**85.9**
TaW	44.35	73	82.2	75	80.0	148	81.1
TaH	29.13	91	85.7	102	81.4	193	83.6

New discriminant functions were also developed for each bone (Tables 6, 7, 8 and 9). For femur, the best percentage of correct classification of sex was obtained with the application of DF1 (91.5%), which requires the nine measurements, whereas the worst results were obtained with DF4 (78.7%), involving the subtrochanteric diameters. In the tibia, the best performance in sex estimation was obtained with the application of DF5 (93.0%), which requires the six measurements, whereas the worst results were obtained with DF8 (83.7%), involving the diameters at the nutrient foramen level. Regarding the calcaneus, the best results were achieved with DF12 (86.6%), requiring the measurement of the body length and the medial breath, whereas the worst results were obtained with DF11 (84.9%), requiring the measurement of the maximum length and the length. Finally, for the talus, the percentages of correct classification were higher for DF14 (88.5%) and lower for DF16 (86.6%). In the first case, the maximum length and the width of the talus are needed and, in the second case, both the width and the height are required.

**Table 6:** Non-standardized coefficients of the new discriminant functions for sex estimation through femur dimensions

FML	DF1	DF2	DF3	DF4
	-0.064			
FPL	0.079	0.018		
FAPMD	-0.08			
FTMD	-0.127			
FAPSD	-0.032			0.36
FTSD	-0.118			0.195
FMC	0.129			
FAPHD	0.171	0.353	0.299	
FVHD	0.187		0.152	
Constant	-21.985	-22.555	-19,352	-15.502
Male Centroid	1.475	1.387	1.297	0.891
Female Centroid	-1.363	-1.282	-1.184	-0.709
% Correct classification for males	95.1	93.8	88.9	76.8
% Correct classification for females	87.9	83.8	84.1	80.6
% Overall Correct classification	**91.5**	**88.8**	**86.5**	**78.7**

**Table 7:** Non-standardized coefficients of the new discriminant functions for sex estimation through tibia dimensions

TML	DF5	DF6	DF7	DF8
	0.148			
TNL	-0.139			
TAPDNF	0.083	0.151	0.114	0.295
TTDNF	0.069			0.177
TMC	0.026		0.036	
TBW	0.175	0.221	0.203	
Constant	-22.647	-20.171	-20.202	-13.916
Male Centroid	1.500	1.382	1.377	1.088
Female Centroid	-1.260	-1.161	-1.147	-0.868
% Correct classification for males	95.2	93.8	93.8	83.9
% Correct classification for females	90.7	85.9	85.9	83.5
% Overall Correct classification	**93.0**	**89.9**	**89.9**	**83.7**

**Table 8:** Non-standardized coefficients of the new discriminant functions for sex estimation through calcaneus dimensions

CML	DF9	DF10	DF11	DF12
	-0.009		0.043	
CL	0.188	0.195	0.227	
CBL	0.020			0.155
CMB	0.180	0.179		0.252
Constant	-20.647	-20.642	-18.846	-19.063
Male Centroid	0.896	0.895	0.855	0.81
Female Centroid	-1.319	-1.318	-1.237	-1.185
% Correct classification for males	87.2	87.2	84.0	86.1
% Correct classification for females	83.0	83.3	85.7	87.0
% Overall correct classification	**85.1**	**85.3**	**84.9**	**86.6**

**Table 9:** Non-standardized coefficients of the new discriminant functions for sex estimation through talus dimensions

TaML	DF13	DF14	DF15	DF16
	0.184	0.252	0.225	
TaW	0.123	0.151		0.176
TaH	0.177		0.251	0.367
Constant	-19.920	-19.476	-18.713	-18.469
Male Centroid	1.166	1.143	1.133	1.100
Female Centroid	-1.119	-1.098	-0.989	-1.070
% Correct classification for males	90.3	90.3	86.5	90.4
% Correct classification for females	85.3	86.7	87.3	82.7
% Overall correct classification	**87.8**	**88.5**	**86.9**	**86.6**

## Discussion

Sex estimation is an important step of biological profile assessment of unidentified individuals in forensic anthropology. Although the pelvis is widely regarded as the best skeletal region for estimating biological sex, several issues may arise. Firstly, results have varied considerably for studies testing the reliability and validity of specific traits and methods. While some have shown high overall accuracy [25], others have demonstrated considerably lower accuracy [26]. In fact, at present, the greatest concern with the morphological methods based on ossa coxae is their low reliability [27]. It should be also highlighted that not all regions of the pelvis yield the same results in terms of accuracy for sex estimation. Overall, the features of the anterior pelvis are more useful than those of the posterior pelvis [28]. However, in forensic contexts, the lower survivability rate of the anterior region is frequently a problem [27]. In fact, there are many cases of incomplete or inexistent remains, as well as instances where the relatively fragile pubic bones have been damaged post-mortem. For those cases where the pelvis is not present for analysis, particularly when remains are scattered or fragmented due to various reasons, namely taphonomic agents, it is very important to have alternative methods for assessing sex on the basis of other parts of the skeleton that may be available for observation and/or measurement [5]. Regarding the skull, traditionally favoured over postcranial bones to estimate sex, more recently research has clearly demonstrated that cranial morphological scoring methods have subjectivity issues [4]. Besides, when forcing the continuous variation observed in cranial traits into discrete ordinal categories, there may be great inter- and even intra-observer errors, and several studies have shown that this can negatively impact sex estimation. For more details see Garvin [29]. On the other hand, recent research has demonstrated that even simple, univariate analyses of single long bone measurements can yield relatively high accuracy levels, besides similar discrimination accuracy between the sexes [8].

In general, methods for estimating sex from the postcranial bones rely on the fact that, within a given population, males tend to be larger than females. However, in order to be possible to estimate sex through metric cut-offs between relatively larger (male) and relatively smaller (female) bones, the degree of sexual differences must be previously known. Since several studies have clearly shown that the expression of human sexual dimorphism is influenced by both genetics and environmental factors (e.g., diet, disease, socioeconomic status), efforts should be made to document population-specific sexual dimorphism and create corresponding methods for sex estimation for each population [9].

Considering the abovementioned need of appropriate sex estimation methods for each population, due to population-specific sexual dimorphism of body shape and size [9, 30], in 2000, Wasterlain [16] developed, based on limb bones dimensions, both cut-off points and discriminant functions to estimate sex in the Portuguese population. However, since these methods were developed on a reference osteological identified collection from the late 19th – early 20th centuries [17], its applicability in forensic contexts needs validation. To this end, the aforementioned methods were applied to an independent sample of skeletons from the 21st Century Identified Skeletal Collection [21].

A total of 22 measurements were performed in several lower limb bones (femur, tibia, calcaneus, and talus). The intra- and inter-observer errors for each anthropometric measurement were evaluated, through the technical error of measurement. Overall, the measurements showed quite low and acceptable values (< 10%) both for intra- and inter-observer errors, confirming their replicability and confidence.

The use of cut-off points in sex estimation presents advantages and disadvantages. While the percentages of correct classification are usually lower than those obtained through the application of discriminant functions, the fact that they are simple and easy to apply in any context, namely in the field, only requiring one measurement and its comparison with the tabulated value, favours its use [11, 16].

In the present study, the vertical diameter of the femoral head (FVHD) proved to be the most effective measurement in estimating the sex of individuals in the CEI/XXI, achieving a percentage of correct classification of 89.8%, followed closely by the talus maximum length (TaML – 87.6%), tibia bilateral width (TBW – 86.2%), antero-posterior head diameter (FAPHD – 85.4%), talus height (TaH – 84.7%), and calcaneus medial breath (CMB – 83.0%). It should be highlighted that, with the exception of the values obtained for talus width (TaW) and femur transverse midshaft diameter (FTMD), which are relatively poor (60% and 70%, respectively), all other cut-off points showed a discriminant capacity above 75%. These results are in broad accordance with those reported by Wasterlain [16] for the original sample. The greater differences were obtained for the tibia antero-posterior diameter at the nutrient foramen, which was reported to have a percentage of correct classification of sex greater in the original method (85.0%) than in the current sample (80.7%), and for the talus width, for which the performance decreased significantly from 78.3% to 60.0%.

Discriminant analysis is a multivariate technique, which is applied when the dependent variable is qualitative and the independent variables are quantitative [24]. The main objective of this type of analysis is to maximize the differences between two groups [31], that is, to choose the variables that distinguish the groups, so that from the characteristics of a new case it is possible to predict to which group it belongs [24]. In other words, this method offers solid and reliable statistical support that, based on various bone measurements, allows one to infer which group (in this case, male or female) an individual belongs to [32].

In this research, the percentages of correct classification obtained with DFs are overall higher that those obtained for cut-off points. In fact, two functions in the femur (DF1) and tibia (DF5) presented correct classifications above 90%, and all others, with the exception of femur DF4 (78.7%), presented correct classifications above 80%. In comparison to the cut-off points results, the performance of discriminant functions increased expressively, despite being already high in the original study.

Although the Wasterlain [16] method has shown satisfactory results when applied to a contemporary Portuguese sample, new cut-off points and discriminant functions were developed in order to promote more accurate sex estimations in forensic cases. Regarding the new cut-off points, the femoral vertical head diameter, the bilateral width of the tibia, the length of the calcaneus, and the maximum length of the talus presented the best results for sex estimation (89.8%, 88.0%, 85.6%, 85.9%, respectively). The new discriminant functions presented correct classification percentages from 78.7% to 91.5% for femur, 83.7% to 93.0% for tibia, 84.9% to 86.6% for calcaneus, and 86.6% to 88.5% for talus. In sum, both the new cut-off points and discriminant functions presented an overall good performance, constituting a reliable method to be applied in forensic cases involving individuals of Portuguese origin.

The differences between the two studies could be related with secular trends between the two samples, mainly due to significant improvements in the diet, health and basic living conditions of the Portuguese population through the 20th century [18–20, 33].

In conclusion, in cases where the pelvis has not been recovered or is not sufficiently well preserved to allow sex estimation, population-specific metric methods based on the post-cranial skeleton are the currently recommended alternative. It was in this context that Wasterlain [16] method emerged, specifically to estimate sex of Portuguese individuals. However, since this method was developed from a Portuguese sample from around 100 years ago, it is crucial that the method be tested and adjusted to the Portuguese forensic context. In the current study, it was shown that, although the Wasterlain [16] method can still present satisfactory results when applied to a contemporary sample, it is recommended to employ the new cut-off points in Portuguese forensic contexts, particularly for the following 12 measurements: femur maximum length, femur physiological length, femur transverse midshaft diameter, femur midshaft circumference, femur vertical head diameter, tibia maximum length, tibia natural length, tibia bilateral width, calcaneus body length, calcaneus medial breath, talus maximum length, and talus height. In turn, the new discriminant functions improved significantly the percentages of correct classification of sex, while using several bone dimensions simultaneously, varying between 78.7% in femur DF4 and 93.0% in tibia DF5. These results are quite positive and, for the most part, align with the principle stated by Berg [15] that a good discriminant function should present percentages of correct classification above 85%. In fact, the only exceptions in the proposed DFs are the already mentioned femur DF4 (78.7%) and the tibia DF8 (83.7%). In both instances, the DFs were developed through the *enter* procedure, forcing the inclusion of the most represented dimensions in the sample, and not necessarily the most discriminant. These DFs may be, however, useful in forensic contexts given their greater probability of being measured. It is important to emphasize that, both in forensic and archaeological contexts, it is rather common that bone preservation and skeletal representativity are relatively poor. Therefore, the availability of a wide range of methods for different preservation conditions is of utmost importance.

In sum, this study presents metric methods based on lower limb bones to estimate sex in Portuguese forensic contexts, providing information on which cut-off points or discriminant functions, those developed by Wasterlain [16] or those created in the present study, to use in each case to obtain the highest accuracy. Both methods present other important advantages, namely the fact that they can be used on different bones with varying degrees of preservation, that they are non-destructive, inexpensive, easy to apply even by less experienced experts and, consequently, present low intra- and inter-observer errors, as clearly demonstrated in the present study.

## Supplementary Information

Below is the link to the electronic supplementary material.

Supplementary Material 1 (DOCX 15.6 KB)

## Data Availability

No datasets were generated or analysed during the current study.
