# Supplementary material for: New method of sex estimation through osteometric analysis of the lower limb for the Portuguese population
Source: Int J Legal Med. 2026 Jul 8;140(6):4473–81. doi: 10.1007/s00414-026-03902-1 (PMC13658320; doi:10.1007/s00414-026-03902-1)
Supplement: Supplementary file 1 [file 414_2026_3902_MOESM1_ESM.docx]

Supplementary table 1. Discriminant functions (DF) proposed by Wasterlain (2000), tested in the present study.

| **Discriminant function (DF)** | |
| --- | --- |
| Femur DF7_W | -0.157*FML + 0.167*FPL + 0.110*FAPMD - 0.103*FTMD + 0.023*FAPSD - 0.026*FTSD - 0.006*FMC + 0.395*FAPHD - 0.021*FVHD - 19.490 |
| Femur DF8_W | 0.133*FAPMD + 0.373*FAPHD - 19.624 |
| Femur DF9_W | 0.079*FAPSD + 0.403*FAPHD - 19.404 |
| Femur DF10_W | 0.076* FAPSD + 0.389*FVHD - 18.867 |
| Femur DF11_W | -0.042 * FTSD + 0.447*FVHD - 18.079 |
| Tibia DF13_W | 0.245*TAPDNF + 0.147*TBW - 17.950 |
| Tibia DF14_W | 0.231*TTDNF + 0.185*TBW - 18.043 |
| Tibia DF15_W | 0.247*TAPDNF + 0.097*TMC - 14.714 |
| Calcaneus DF19_W | 0.047*CML + 0.221*CL - 0.170*CBL +0.271*CMB - 20.740 |
| Calcaneus DF20_W | 0.260*CL - 0.161*CBL + 0.274*CMB - 20.590 |
| Calcaneus DF21_W | 0.141*CL + 0.268*CMB - 20.524 |
| Talus DF16_W | 0.272*TaML + 0.019*TaW + 0.236*TaH - 21.283 |
| Talus DF17_W | 0.278*TaML + 0.249*TaH - 21.210 |
| Talus DF18_W | 0.108*TaW + 0.519*TaH – 19.290 |
